# The voltage-gated proton channel hHv1 is functionally expressed in human chorion-derived mesenchymal stem cells

**DOI:** 10.1038/s41598-020-63517-3

**Published:** 2020-04-28

**Authors:** Beáta Mészáros, Ferenc Papp, Gábor Mocsár, Endre Kókai, Katalin Kovács, Gabor Tajti, Gyorgy Panyi

**Affiliations:** 1 Department of Biophysics and Cell Biology, Faculty of Medicine, University of Debrecen, Life Science Building, Debrecen, Egyetemter 1 Hungary H-4032; 2 Department of Medical Chemistry, Faculty of Medicine, University of Debrecen, Life Science Building, Debrecen, Egyetemter 1 Hungary H-4032; 3 MTA-DE Cell Biology and Signaling Research Group, University of Debrecen, Life Science Building, Debrecen, Egyetemter 1 Hungary H-4032

**Keywords:** Ion transport, Mesenchymal stem cells

## Abstract

The voltage-gated proton channel Hv1 is widely expressed, among others, in immune and cancer cells, it provides an efficient cytosolic H^+^extrusion mechanism and regulates vital functions such as oxidative burst, migration and proliferation. Here we demonstrate the presence of human Hv1 (hHv1) in the placenta/chorion-derived mesenchymal stem cells (cMSCs) using RT-PCR. The voltage- and pH-dependent gating of the current is similar to that of hHv1 expressed in cell lines and that the current is blocked by 5-chloro-2-guanidinobenzimidazole (ClGBI) and activated by arachidonic acid (AA). Inhibition of hHv1 by ClGBI significantly decreases mineral matrix production of cMSCs induced by conditions mimicking physiological or pathological (inorganic phosphate, Pi) induction of osteogenesis. Wound healing assay and single cell motility analysis show that ClGBI significantly inhibits the migration of cMSCs. Thus, seminal functions of cMSCs are modulated by hHv1 which makes this channel as an attractive target for controlling advantages/disadvantages of MSCs therapy.

## Introduction

Mesenchymal stem cells (MSCs) are multipotent cells with intensive proliferative capacity and ability to differentiate into various cell types (osteoblasts, chondroblasts, myocytes, adipocytes etc.)^1,2^. MSCs were originally isolated from bone marrow, and were found later in numerous organs and tissues, including adipose tissue, periosteum, synovial membrane, articular cartilage, umbilical cord and placenta, among others^1,3^. The use of MSCs is a novel therapeutic strategy for regenerative medicine^4,5^, however, their application is not limited to repairing and replacing the impaired organs, rather, the immunomodulatory and anti-inflammatory properties^5–9^ of MSCs are also important^10,11^. Placenta-derived mesenchymal stem cells (cMSCs) possess excellent immunoregulatory properties, therefore, the chorionic plate of the placenta may be an attractive source for stem cells to be used in cell therapy and tissue engineering^3,12^. In most cases, systemic delivery is preferred for the clinical applications which requires the homing and migration of MSCs to the target tissue. In line with these MSCs have a capacity to migrate into the injured and inflamed environment^13,14^.

Bioelectric signaling and pH regulation via ion channels and pumps are known to play a role in a wide range of cell functions, including cell proliferation, migration, differentiation, apoptosis but this aspect of stem cell biology seems to be poorly understood^10,15^. Multiple ion channels were reported earlier to be present in human MSCs, for example K^+^channels, Na^+^and Cl^−^ channels^16,17^, but data are missing in the literature for the existence of the human voltage-gated proton channels (hHv1) in mesenchymal stem cells. At the same time hHv1 channels are widespread; they can be found in various mammalian cells^18–24^, such as macrophages^25^, B-lymphocytes^26,27^, oocytes^28^, osteoclasts^29,30^, skeletal muscle cells^31^, as well as cancer cells^32,33^, for example in malignant B-cells^22^, Jurkat cells^18^, and glioblastoma multiforme^21^.

Voltage-gated proton channels have characteristic biophysical properties, e.g., they are highly selective for protons and the channels have extremely low single-channel conductance^19^. Their gating is voltage-and pH dependent: changing of the intra- or extracellular (pH_i_ or pH_o_, respectively) pH by one unit shifts the voltage-dependence of gating by 40 mV^34^. In most species they conduct non-inactivating outward current only. The function of hHv1 strongly depends on the temperature as well^35,36^. As for pharmacology, Hv1 can be inhibited by Zn^2+^ ^37,38^, ClGBI^39^ and a peptide inhibitor (Corza6)^40^, and the channels can be activated by arachidonic acid, however, this latter effect requires PKC activation^41–43^. The function of Hv1 is associated with many cellular processes^19^, such as migration^20^, proliferation^44^ and apoptosis^18,21^, which are highly relevant to the physiology and pathophysiology of MSCs^18–21^.

Based on the widespread expression of Hv1 and its versatile physiological functions we hypothesized that this channel may be present in MSCs as well. To confirm this hypothesis, we demonstrated the expression of Hv1 mRNA transcripts in cMSCs using RT-PCR. We also measured the native proton current in cMSCs using the whole-cell patch-clamp technique and found that its biophysical and pharmacological characteristics (including pH- and voltage-dependence, ClGBI sensitivity, activation by AA) were consistent with the properties of the Hv1 channel. As for the physiological function of the channel, we found that the activity of Hv1 influences cell viability and mineral matrix formation during physiological and pathological mineralization. Moreover, blocking of hHv1 inhibited the motility of these cells. We propose that hHv1 might be a new target or control point in the regulation of therapeutic application of MSCs.

## Results

### Expression of human voltage-gated proton channel transcripts in cMSCs

**Figure 1:**
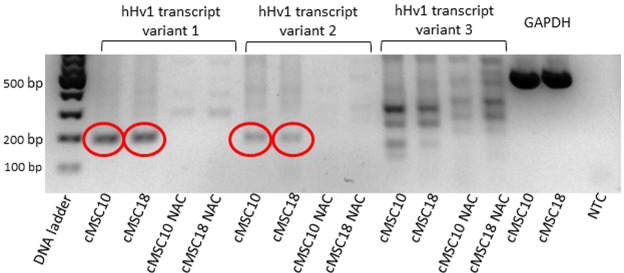
hHv1 transcript variants 1 and 2 are detected in cMSCs from placenta donor 10 and 18. Identification of hHv1 mRNA was carried out by means of RT-PCR, using intron spanning primers to confine the distinct transcript variants. Red circles on the gel electrophoresis photos highlight the presence of the transcripts of hHv1 variant 1 (PCR product is 190 bp, left set) and hHv1 variant 2 (PCR product is 183 bp, middle-left set) in cMSCs isolated from placenta donors 10 (shown as cMSC10 in the figure) and 18 (shown as cMSC18 in the figure), as indicated. We did not detect the transcript variant 3 (missing 568 bp long PCR product, middle-right set). The PCR was validated using GAPDH and NTC (non-template control) controls (right set). The NAC bands (no amplification control) were obtained in the absence of the reverse transcriptase enzyme. NAC controls showed that genomic hHv1 DNA was not amplified in the positive samples. Full-length gels are presented in Supplementary Fig. S9.

RT-PCR was used to characterize the expression of the mRNA encoding the hHv1 channel. Based on the GenBank database the hHv1 channel is encoded by the HVCN1 gene with three distinct transcript variants. Accordingly, we have designed intron-spanning primers to identify and confine the three transcript variants. Figure 1 shows that transcript variants 1 and 2 were detected in the chorion derived MSCs from two different placentas. We carried out this analysis with cMSCs isolated from two other placenta donors as well (Supplementary Fig. S1), our results were the same in all placentas examined. The expression of transcript variants 1 and 2 was maintained during the 21-day exposure of cMSCs to the cocktail inducing osteogenic differentiation (Supplementary Fig. S1, see later for osteogenic induction). Transcript variants 1 (NM_00104017.1) and 2 (NM_032369.3) encode the same, longer hHv1 protein isoform, whereas transcript variant 3 (NM_001256413) results in the shorter isoform. Our results demonstrated the expression of transcript variants 1 and 2 in mesenchymal stem cells, which predicts the presence of the longer, common isoform of the hHv1 protein in these cells. The presence of transcript variants 1 and 2 in differentiated cells as well suggest that the expression pattern of hHv1 isoforms does not depend on the differentiation status of the cells.

### Modulation of the voltage-dependent gating of the current by extracellular pH

**Figure 2:**
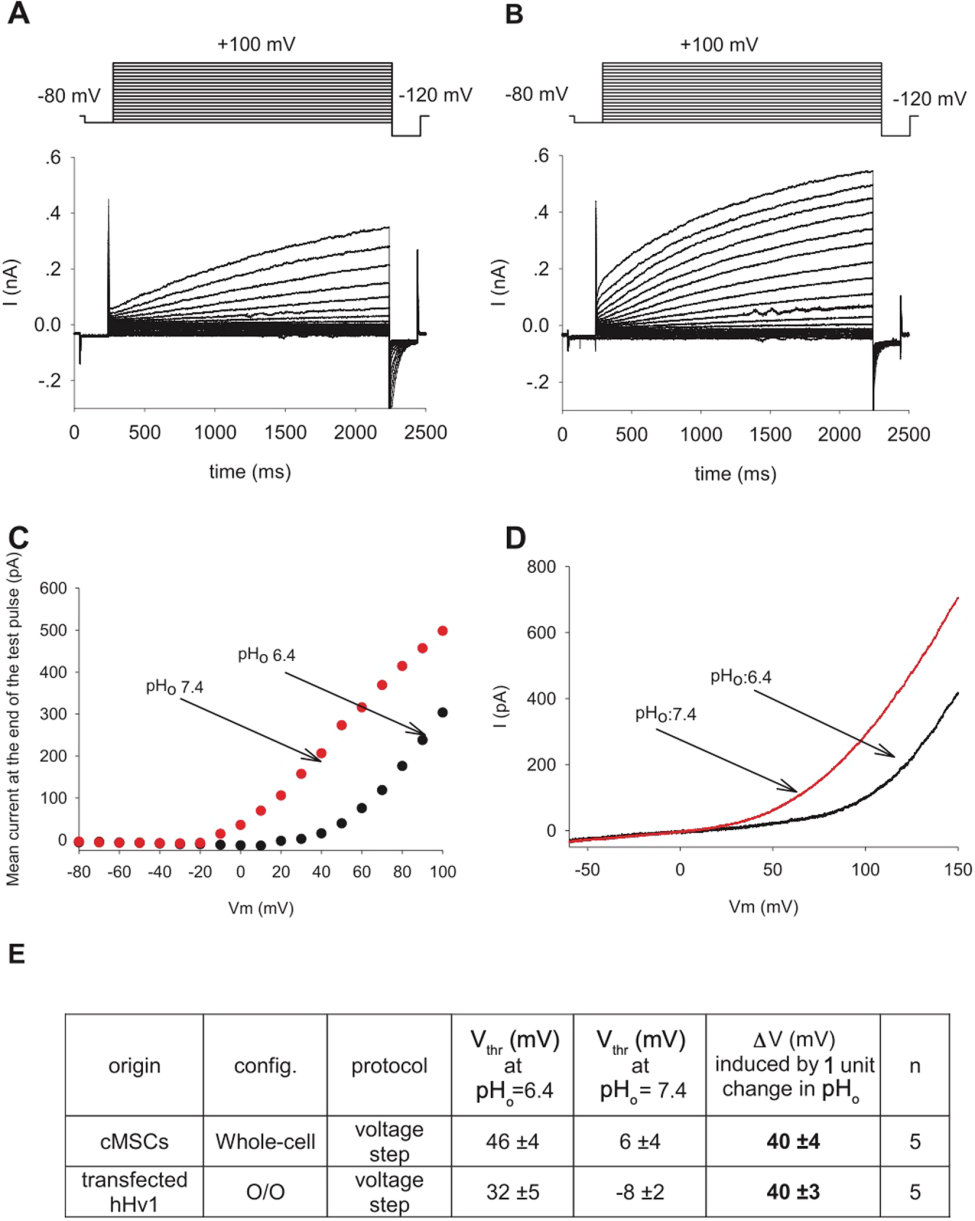
Voltage- and pH-dependent gating of the hHv1 in mesenchymal stem cells. Increasing extracellular pH by one unit shifts the voltage-dependence of activation of Hv1 channels by about 40 mV toward more negative voltages. A, Whole-cell currents in a cMSC (donor 10) using pH_o_ = 6.4 bath and pH_i_ = 6.18 pipette filling solutions. The cell was held at −80 mV holding potential, test pulses were delivered ranging from −80 mV to +100 mV with 10 mV increment every 20 s followed by a short hyperpolarization to −120 mV(inset) before returning to the holding potential. The duration of the test pulses was 2000 ms. B, Whole-cell currents of the same cell as in panel A but using pH_o_ = 7.4 bath and pH_i_ = 6.18 pipette filling solutions. For the composition of the recording solutions see Materials and Methods. C, Current- voltage relationships constructed from the records shown in A and B after leak subtraction. Data points were determined as the average of the last 20 points at the end of the depolarizing pulses at the indicated test potentials. Red and black circles indicate the magnitude of the currents in pH_o_ = 6.4 and pH_o_ = 7.4, respectively. D, Whole-cell currents evoked by voltage ramps at different pH_o_. The same cMSC as in panels A and B, red and black traces correspond to records at pH_o_ = 7.4 and pH_o_ = 6.4, respectively, as indicated also by the arrows. The holding potential was −60 mV, the membrane potential was changed at a constant rate from −60 mV to +150 mV in 1000 ms, every 20 s. E, Summary of the modulation of the voltage-dependent gating by pH in cMSCs and transfected cells. Config. whole-cell and O/O corresponds to whole-cell and outside-out configuration of patch-clamp, respectively, protocol IV stands for voltage step protocol as shown in panels A and B, n indicates the number of experiments. ∆V (mV) is the change in the threshold potential (V_thr_).

Figure 2A shows whole-cell currents of a cMSCs recorded at pH_i_ = 6.18 in the pipette filling solution and pH_o_ = 6.4 in the extracellular solution at increasing depolarizations (inset). The recording solutions lacked conventional permeating cations and contained reduced Cl^−^ concentration to eliminate outward currents other than the proton current (see Materials and Methods and Discussion). Upon depolarization exceeding +50 mV robust voltage- and time-dependent whole-cell currents were recorded (Fig. 2A). Larger depolarizations evoked larger currents with faster activation kinetics, albeit, the current activates slowly even at large depolarizations, it does not saturate during the 2000-ms-long pulse, and inactivation of the current was not observed. The extracellular solution was then switched to pH_o_ = 7.4 (Fig. 2B), which increased the currents at identical test potentials and sped up the activation kinetics. The magnitudes of the currents at the end of the depolarizing pulses were determined at pH_o_ = 6.4 and pH_o_ = 7.4 and plotted as function of the test potential in Fig. 2C. Comparison of the two datasets shows that the data points obtained in the pH_o_ = 7.4 solution are shifted to hyperpolarized potentials as compared to pH_o_ = 6.4; one-unit shift in pH_o_ shifted the current-voltage relationship by ~40 mV in this cell. The V_thr_ (threshold potential at which Hv1 starts to open, see Methods, Supplementary Fig. S2) was +46 ± 4 mV at pH_o_=6.4, and V_thr_ = +6 ± 4 mV at pH_o_ = 7.4 in cMSCs, which corresponds to a 40.0 ± 4 mV (n = 5) voltage shift (Fig. 2E). The pH_o_-induced shift in the current-voltage relationship is also demonstrated using voltage-ramps. Figure 2D shows voltage ramp-induced (from −60 to +150 mV) currents at pH_o_ = 7.4 and at pH_o_ = 6.4 in a cMSC, the shift in the I-V curve obtained in this way is also ~40 mV. Statistical analysis of the voltage-ramp evoked currents shows a 37.6 ± 1.7 mV shift in the activation threshold (n = 10) upon changing the extracellular pH (∆pH_o_ = 1). Similar experiments were conducted in HEK cells expressing the hHv1 channel (Supplementary Fig. S3). The shifts in the threshold potentials induced by ∆pH_o_ = 1 were 40.0 ± 3.0 mV and 42.8 ± 3.6 mV for voltage-step and voltage-ramp protocols, respectively. V_thr_ values were +32 ± 5 mV and −8 ± 2 mV (n = 5),using pH_o_ = 6.4 and pH_o_ = 7.4 solutions, respectively, for the hHv1 currents measured in outside-out configuration of the patch-clamp in HEK.

### Arachidonic acid enhances and ClGBI inhibits the hHv1 conductance in cMSCs

**Figure 3:**
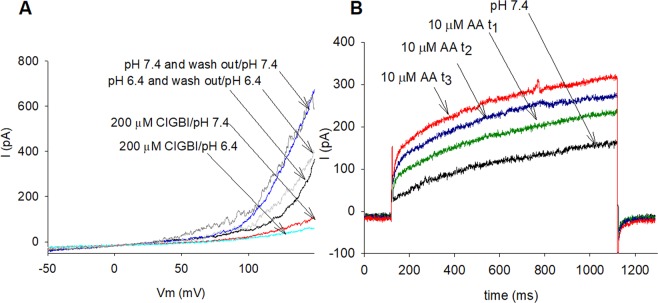
Sensitivity of hHv1 to ClGBI and Arachidonic Acid (AA) in cMSCs. The Hv1 current is activated by micromolar concentrations of arachidonic acid (AA) in the extracellular solution^41,43^. This feature is shown in Fig. 3B, where currents evoked by voltage steps to +100 mV are shown in control solution at pH_o_ = 7.4 and after the perfusion of the recording chamber with the same solution but containing 10 µM AA. The traces obtained at three different time points during the experiment clearly indicate the time-dependent enhancement of the current by AA. The current amplitude at +100  mV increased 1.43 ± 0.16-fold (n = 3) at t_1_ = 3 min, 1.77 ± 0.14-fold (n = 3) at t_2_ = 3.3 min and 2.14 ± 0.18-fold (n = 3) at t_3_ = 3.5 min, as compared to the control current. The enhancement of the current was reversible upon perfusing the recording chamber with AA-free extracellular solution (Supplementary Fig. S5).

One of the pharmacological hallmarks of the Hv1 channel is its sensitivity to the guanidine-derivate blocker ClGBI (5-chloro-2- guanidinobenzimidazole). Figure 3A shows the whole cell currents evoked by voltage-ramps in a cMSC at pH_o_ = 6.4 and in the absence and presence of 200 µM ClGBI. This concentration of CIGBI inhibited 83–91% of the current measured at the end on the ramp at +150 mV (pH_o_ = 7.4: 83 ± 1.4%, n = 7; pH_o_ = 6.4: 91 ± 1.1%, n = 7), the block of the current was significantly different at the two pH_o_values (t-test, p = 0.002, n = 7). The block was reversible, the current returned to the control by perfusing the recording chamber with drug-free solution. These properties of the block agree well with the literature ((K_d_was 26.3 ± 2.2 µM^39^) and with our data in HEK cells transfected with hHv1(Supplementary Fig. S4).

A, Inhibition of the hHv1 current by ClGBI. Whole-cell currents evoked by voltage-ramps in the presence and absence of ClGBI at different pH_o_. The holding potential was −60 mV, the membrane potential was changed at a constant rate from −60 mV to +150 mV in 2000 ms, every 20 s. Control records were obtained at pH_o_ = 6.4 (black) and pH_o_ = 7.4 (blue) and in the presence of 200 µM ClGBI at pH_o_ = 6.4 (cyan) and pH_o_ = 7.4 (red). 92% and 86% of the current at +150 mV was blocked at pH_o_ = 6.4 and pH_o_ = 7.4, respectively. Wash-out traces are shown for pH_o_ = 6.4 (gray) and for pH_o_ = 7.4 (dark gray). B, Enhancement of the hHv1 current by AA. The currents were measured in whole-cell configuration. Depolarizing voltage pulses (+100 mV, 1 s in duration) were applied every 30 s from holding potential −80 mV. Control record was obtained at pH_o_ = 7.4 (black) then the perfusion of the recording chamber was changed to the same solution containing 10 µM AA and the currents were recorded at t_1_ = 3 min (green), t_2_ = 3.3 min (blue) and t_3_ = 3.5 min (red). The current was enhanced 1.4-fold, 1.7-fold and 2-fold at t_1_, t_2_ and t_3_, respectively in this record.

### The hHv1 blocker ClGBI reduces the viability of cMSCs

**Figure 4:**
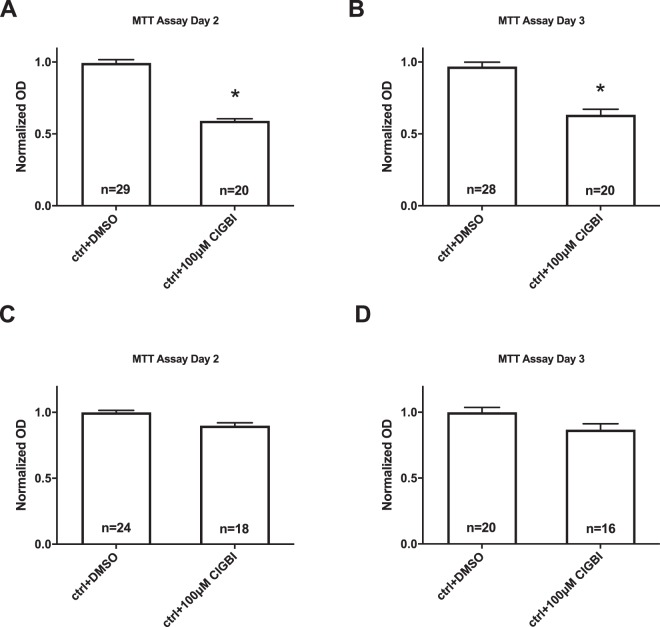
Effect of ClGBI on the viability of Hv1 expressing (cMSCs) and non-expressing (COS-7) cells. A, B Reduction in the cell viability of cMSCs in the presence of the Hv1 blocker ClGBI. Normalized OD was calculated as A/A_ctrl_ where A and A_ctrl_ are the absorbances of a given sample and that of cMSCs cells in the presence of DMSO control, respectively. ClGBI was added to the cell culture medium in the indicated concentrations. OD determinations were done on samples harvested on day 2 (A) and day 3 (B). Significant differences (p < 0.05) are indicated by asterisks, bars and error bars indicate mean ± SEM for the indicated number of experiments. C, D ClGBI does not affect the viability of COS-7 cells. The same set of experiments as in A and B were repeated in COS-7 cells, variables are the same as above. Similarly, DMSO treatment was used as vehicle control, results were normalized to COS-7 cells treated with DMSO.

A recent publication reported that inhibition of hHv1 induced apoptosis in Jurkat cells^18^, therefore, we tested if block of hHv1 in cMSC influences cell viability measured by the MTT reduction assay. The measurement is based on the conversion of MTT (Thiazoly Blue Tetrazolium Bromide) to formazan by NAD(P)H-dependent oxidoreductase enzymes of viable cells only. Therefore, the amount of formazan dye formed directly correlates to the number of metabolically active cells in the culture^45^. Figure 4A,B show that ClGBI (100 µM) reduced the viability of cMSCs on day 2 (A) and day 3 (B) of the treatment. The control solution contained DMSO (ctrl + DMSO) as the ClGBI stock solution was prepared in DMSO. The reduction in the cell viability was estimated by the decrease in the normalized OD (see Materials and Methods). A similar set of experiments was conducted in COS-7 cells (Fig. 4C,D) which do not express Hv1 proton channels^46^. We found that the cell viability was not affected by 100 µM ClGBI in COS-7 regardless of the day of the determination, i.e., cells lacking the Hv1 proton channel were insensitive to the inhibitor of the voltage-gated proton current.

### Mineral matrix production of cMSCs is inhibited by blocking of hHv1

**Figure 5:**
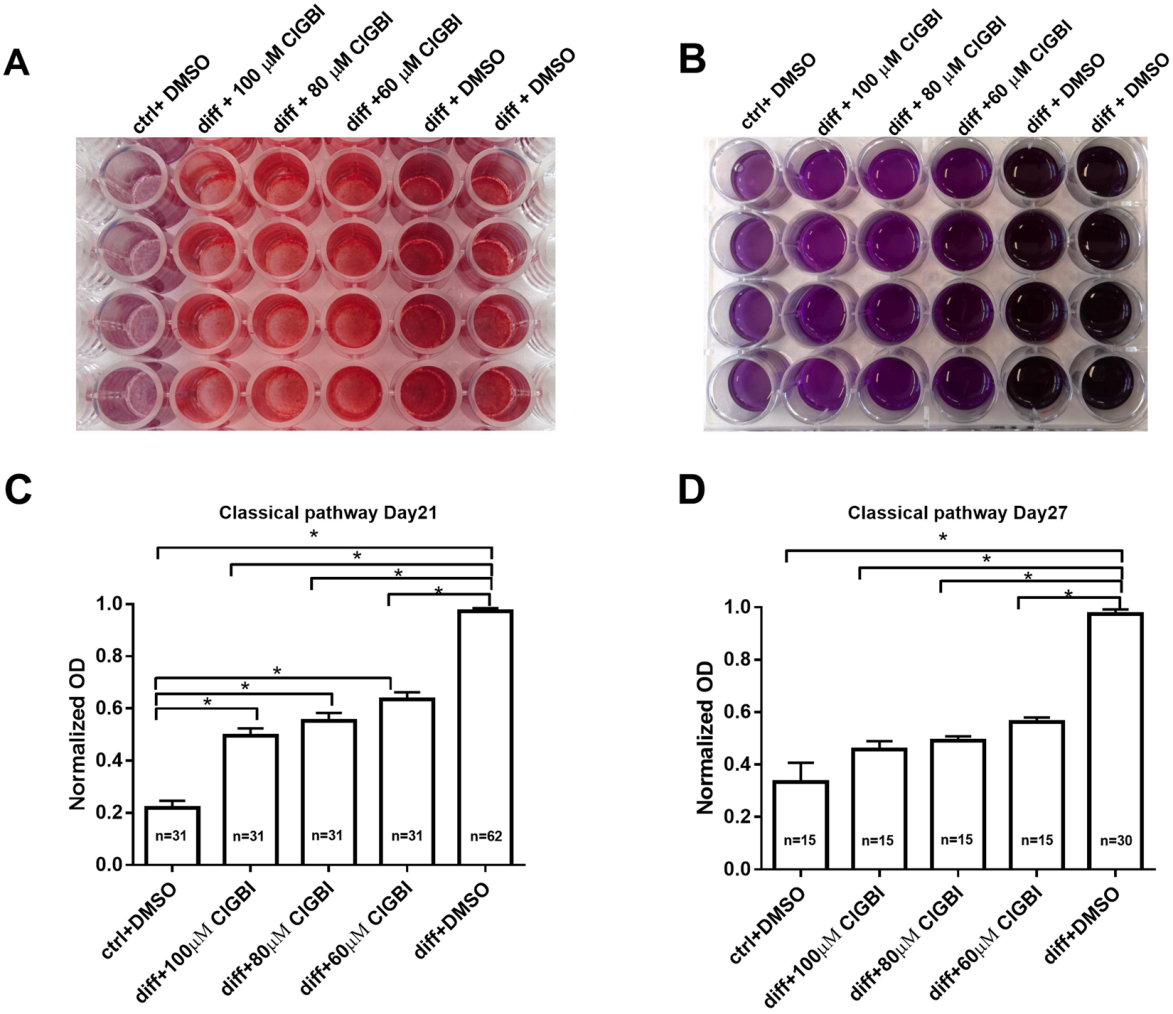
Sensitivity of the classical pathway-induced mineral matrix production to inhibition of hHv1proton channels. A-B, Mineralization of the ClGBI-treated and non-treated cMSCs using Alizarin Red staining (A) and upon dissolving the Alizarin Red S-calcium complexes in CPC (B). Labels indicate cMSC cultures treated with ctrl + DMSO: no differentiation cocktail, DMSO added as vehicle control for ClGBI; diff + ClGBI: induced by the cocktail for the classical pathway and treated with indicated concentrations of ClGBI; diff + DMSO: induced by the cocktail for the classical pathway and added DMSO as vehicle control for ClGBI. Staining was done on day 27. C-D: Quantification of the mineral matrix production on the Day 21 (C) and Day 27 (D). Normalized mineralization was calculated as A/A_DMSO_ where A and A_DMSO_ are the absorbances for a given sample and that for differentiation-induced cells in the presence of DMSO (vehicle control), respectively. Data were analyzed using One Way Analysis of Variance on Ranks statistical test, *p < 0.05.

**Figure 6:**
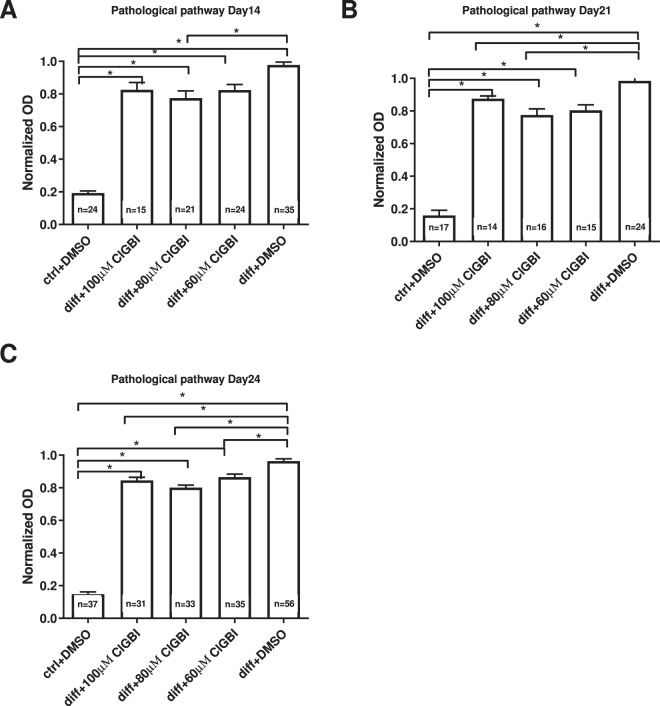
Sensitivity of the pathological pathway-induced mineral matrix production to inhibition of hHv1proton channels. Mineralization of cMSCs was induced in via the pathological pathway using inorganic phosphate (Pi) and mineralization was assayed on Day 14 (A), Day 21 (B) and Day24 (C) using Alizarin Red. ClGBI was added to differentiation-induced cultures (diff + ClGBI) in the indicated concentrations and DMSO was used as vehicle control for ClGBI (diff + DMSO). ctrl + DMSO sample was treated with DMSO only in the absence of inorganic phosphate. Normalized mineralization was calculated as A/A_DMSO_ where A and A_DMSO_ are the absorbances for a given sample and that for differentiation-induced cells in the presence of DMSO (vehicle control), respectively. Data were analyzed using ANOVA statistical test, *p < 0.05.

Osteogenic induction (including mineral matrix production) of cMSCs was performed using dexamethasone, ascorbic acid, β-glycerophosphate and vitamin D_3_ treatment, referred to as “classical” pathway of osteogenic induction^47,48^. Osteogenic differentiation of cMSCs was followed and quantitatively analyzed using Alizarin Red staining (Fig. 5). To test the hypothesis that inhibition of hHv1 modulates osteogenic differentiation of MSCs the inhibitor of hHv1, ClGBI was added to the differentiation medium in various concentrations. Figure 5A shows the Alizarin Red staining of the cultures on day 27. The darkest red wells on the right indicate strong mineral matrix formation in vehicle controls (diff + DMSO). The progressively lighter colors with increasing ClGBI concentrations correspond to a dose-dependent inhibition of mineralization by ClGBI. The lightest red colors on the left show the differentiation control where the differentiation cocktail was omitted from the medium only DMSO was added as vehicle control for ClGBI. The hydroxyapatite-Alizarin Red complexes were dissolved in CPC (Fig. 5B) and the optical density was determined. The inhibition of the mineral matrix production is clearly reflected in the normalized optical densities of the different samples shown in Fig. 5C,D. The inhibition of mineral matrix formation increases with increasing ClGBI concentration, the normalized optical densities are significantly smaller at higher ClGBI concentrations (*p < 0.05, One Way Analysis of Variance on Rank statistics). The extent of inhibition was statistically the same on day 27 as on day 21 at identical ClGBI concentrations (*p > 0.05, t-test or Mann-Whitney Rank Sum Test). We also examined the effect of Hv1 blockers on the inorganic phosphate (Pi)-induced mineralization of MSCs, which process may be similar to the pathological mineralization therefore referred to as such below. The same set of experiments were conducted as above, except the duration of the experiments, which were 14, 21 and 24 days following pathological induction of mineral matrix formation. Figure 6 shows the time and concentration dependence of the effect of ClGBI on this process. Regardless of the duration of the experiment (i.e. the time point of the readout) ClGBI induced a 10–20% inhibition in the mineral matrix production. Furthermore, the inhibition lacks clear dose-dependence: 100 µM ClGBI had smaller (day 14) or equivalent (day 21) effect as 60 µM ClGBI.

### Migration of MSCs is inhibited by blocking of hHv1

**Figure 7:**
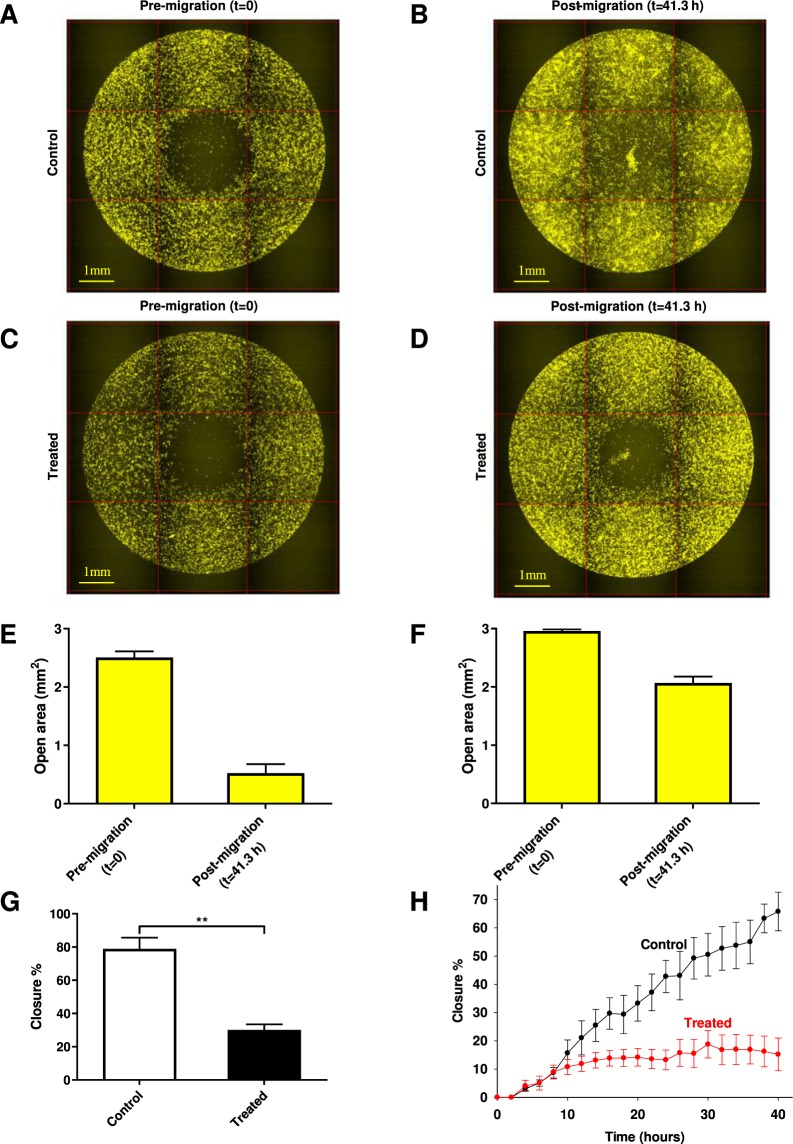
Representative images of the wound healing assay and the high content screening of closure responses using Oris Pro assay. Representative CM-Dil-20 stained images of cMSCs are shown at *t* = 0 for non-treated (A) and ClGBI-treated (100 µM) wells (B). Images were taken immediately following cell seeding. The final images of the closure areas (*t* = 41.3 h) are in panel C (control) and D (treated) respectively. Intensities were rescaled, scale bar represent 1 mm. Diagrams present open area (cell free zone) of ClGBI-treated (100 µM, F) and non-treated (control; E) cMSCs at *t* = 0 and *t* = 41.3 hours. Each bar represents the average and SEM of 7 wells (n = 7). G, Comparison of closure responses of MSCs following treatment with ClGBI (100 µM) over 41.3 hours versus control non-treated MSCs. Closure% = [(initial cell free are − cell free are at a given time pont)/initial cell free area] * 100). ***p < 0.0001 where indicated versus non-treated control. H, Time-dependence of the closure responses of MSCs in the absence (Control, black) and presence of 100 µM ClGBI (Treated, red). Each time point represents the average percent closure and SEM of samples from 3 different placenta donors.

It has been reported that voltage-gated H^+^channels are involved in the regulation of migration of cancer and immune cells^21,24,32,33^. Based on this we hypothesized a similar effect in mesenchymal stem cells, therefore, we tested the effect of 100 µM ClGBI on the migration of cMSCs. We used the Oris Pro Biocompatible Gel to form a cell-free zone on a cell culture plate to result in a standardized wound-healing assay. The pre-labelled cells were seeded into the plate and monitored continuously over 41.3 hours using high content screening microscopy, and we quantified the closure of the initially acellular zone in each well. The initial cell free area is the cell free exclusion zone at the beginning of experiment (Fig. 7A,B), this area practically vanished by the end of the experiment (t = 41.3 h) in control condition (Fig. 7C). The initial cell free area reduced slightly in 41.3 h when 100 µM ClGBI was present (Fig. 7D). Cell fee areas are quantitatively displayed for control and 100 µM ClGBI-treated samples in Fig. 7E,F, respectively. Closure% of the cell free zone was used to assess wound-healing and calculated as follows: closure % = [(initial cell free area − cell free are at a given time point)/initial cell free area] * 100. A higher closure % indicates a smaller cell free zone in the wound. Figure 7G shows the comparison of the average closure% (±SEM) for control and 100 µM ClGBI-treated samples at 41.3 h. Measurements were carried out in duplicates using cMSCs from three different placenta donors, however, Fig. 7A–G demonstrates the data obtained in one experiment for one donor. Figure 7H presents the time-dependence of the reduction in the closure % obtained from pooled data (all experiments on all samples of the 3 donors). Figure 7H shows that control and ClGBI-treated samples display similar closure % values during the first 8 hours of the assay. Thereafter, the closure% in the presence of 100 µM ClGBI is significantly reduced as compared to control: the open area continuously decreases in the control samples whereas it remains constant in the ClGBI-treated samples. Time-dependence of the closure % responses, calculated individually for each sample, showed similar characteristics to the pooled ones (data not shown).

**Figure 8:**
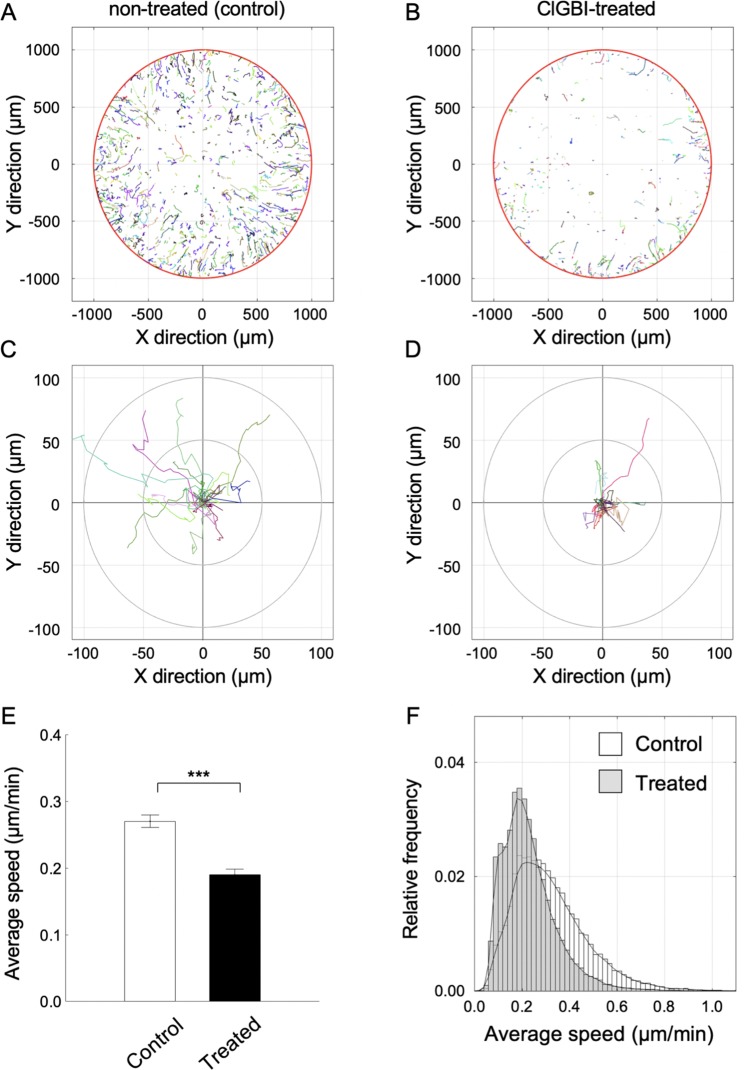
Single cell motility analysis demonstrates anti-migratory activity of ClGBI. Representative cell migration tracks over a period of 41.3 hours are shown for non-treated (A) and ClGBI-treated (100 µM) wells (B). The initial, cell free circular region of the center of the wells was used as a spatial filter for constructing the cell trajectories. Each colored track represents a different cell. Rose plots illustrate the motility patterns of individual cells in ClGBI-treated (D, 100 µM) and non-treated (C) samples. Twenty representative trajectories within the initial cell free region with 20 steps were used to construct the rose plots, each track represents the trajectory of a single cell over a period of 6.6 hours. Migration ability of cells was quantified by calculating the average speed of the cells along each trajectory. Means of cellular speeds of treated (filled bar) and non-treated (empty bar) tracks are summarized in panel E (*n* = 35–40), error bars are SEM values, data were compared with two tailed Kolmogorov-Smirnov tests, ***p < 0.0001. (F) The distributions of the average speeds for all treated (grey bars) and not-treated (empty bars) tracks (*N* > 20,000). The probability density histograms were estimated (continuous black lines) by calculating the kernel smoothing functions of speed data.

ClGBI treatment inhibits the closure of the cell free zone during the time course of the experiment. The wound healing assay, however, does not discriminate among anti-migratory from anti-proliferative or apoptotic effects of the drug. To address directly the effect of ClGBI on migration we carried out single cell analysis of the cell motility (Fig. 8). The assays were performed in 2% serum-containing media, which inhibits cell proliferation and the consequent reduction of the cell-free zone due to increase in the cell number. We analysed the trajectories (Fig. 8A,B) of cMSCs in the center region (initially cell-free zone) of the Oris Pro Assay plate. The visual comparison of the rose-plots obtained in the absence (Fig. 8C) and presence of 100 µM ClGBI (Fig. 8D) qualitatively shows that ClGBI-treatment reduces the motility of cMSCs. Quantitative analysis of the trajectories was obtained by determining the average speed of migration, which was significantly reduced in the presence of ClGBI (Fig. 8E). The frequency histogram (Fig. 8F) shows the reduction in the average speed of trajectories at the population level in the presence of ClGBI. The shift of the histogram to lower average speed values in the presence of ClGBI suggests the involvement of hHv1 in the regulation of the migration ofcMSCs.

## Discussion

A TTX sensitive voltage-gated sodium current, Clcn3 chloride channels, KCa1.1, Kv10.1, Kir2.1, Kir6.1, Kir6.2, KCa3.1 potassium channels and L-type Ca channel were identified in MSCs from different sources including bone marrow and human umbilical cord vein^16,49–51^. These channels participate in the regulation of proliferation^52^, migration^53^, osteogenic^51,52^ and adipogenic differentiation^51–53^. Furthermore, membrane potential (V_m_) can be a tractable control point for modulation of stem cell differentiation in a bi-directional fashion^10,15^. In this study, we detected transcript variants 1 and 2 of the HVCN1 gene using RT-PCR, which predicts the presence of the common, longer isoform of the human voltage-gated proton channels in cMSCs. The longer isoform is more widespread than the shorter, the latter was identified in malignant B-cells^20^ only.

The main properties of the voltage-gated proton channels are the followings^17,24,34^: modulation of the voltage-dependent gating by pH; the absence of inactivation; inhibition of the current by Zn^2+^ and 5-chloro-2- gunidinobenzimidazole (ClGBI), whereas arachidonic acid (AA) enhances the Hv1 current. To confirm the functional expression of the hHv1 in these cells we carried out electrophysiological measurements. Changing of pH_o_ by one unit shifts the conductance-voltage relationship by ~40 mV, which is a hallmark of the Hv1 ion channels^19^. For the objective determination of the threshold potential of activation we have applied mathematical criteria for both voltage-ramp and voltage-step-based records (Supplementary Fig. S2). Data presented in the manuscript reflect V_thr_ values obtained using that approach. We obtained the same magnitude of shift in V_thr_ of the current in cMSCs and HEK293 cells transfected with hHv1, although the threshold potential was different for the two cell types. Previous publications have also reported that threshold potential for current activation may vary from cell to cell^17,41^. The resting membrane potential of MSCs is more depolarized than in mature cells^3,10^, and there are differences between ‘phagocyte’ and ‘epithelial’ type of native proton conductances^19,23,38^. A higher threshold potential could serve the special electrophysiological needs of mesenchymal stem cells.

There are two pharmacological inhibitors of hHv1 most commonly used for the identification of hHv1. Of these 200 µM ClGBI resulted in 83–91% block of the whole-cell current in agreement with the literature (K_d_ = 26.3 ± 2.2 µM)^34^. The other well-known inhibitor is Zn^2+^ that fully inhibits hHv1 at 100 μM concentration^37,38^. However, we were unable to use Zn^2+^ for positive identification of the current. Our pilot experiments showed the expression of robust Cl^−^currents in cMSC (Meszaros *et al*., in preparation) which were eliminated using low Cl^−^concentration extra- and intracellular solutions in the current study. The substitute for Cl^−^ was aspartate, which complexes Zn^2+^ ^54^ and thus, reduces the potency of Zn^2+^ in inhibiting hHv1 (Supplementary Fig. S6). The two critical residues, His140 and His 193^19,37,38^, that are required for high-affinity Zn^2+^ binding to hHv1, are present in the hHv1 transcripts from cMSCs (Supplementary Fig. S7). The presence of the His residues predicts Zn^2+^ sensitivity of the hHv1 current in cMSCs as well.

Voltage-gated proton channels are activated by arachidonic acid (AA)^41^, an ω-6 polyunsaturated fatty acid. Accordingly, we tested the sensitivity of the whole-cell current of cMSCs to AA. 10 µM AA significantly enhanced the current in a time-dependent, reversible manner (Fig. 3B). AA also activates potassium current trough KCa1.1, however, there was no potassium ion neither in the intracellular or the extracellular solution thereby excluding the contamination of the whole-cell current by KCa1.1. The co-existence of AA-induced activation of both KCa1.1 and the hHv1 current was also tested using K^+^-containing solutions (Supplementary Fig. S8). KCa1.1 was first activated by 30 µM AA then inhibited fully by paxillin (the subunit composition-independent inhibitor of the KCa1.1 channels). In the continuous presence of paxillin and AA a whole cell outward current resembling hHv1 current reappeared in ~5 min. Arachidonic acid can be generated endogenously in the human cells (e.g. released from cell membranes of phagocytes by phospholipase A2 during inflammatory reactions). The liberated AA could regulate various biological processes including reactive oxygen species production by NADPH oxidase. The potentiation of the whole-cell current by AA suggests that perhaps, endogenous AA regulates the Hv1 channel activity in cMSCs and the relationship between the immune cells and MSCs may be strong, as many publications described it earlier^1,3,55–57^. It is also unclear if the expression of hHv1 is a property exclusive to cMSCs, or, perhaps, hHv1 can also be expressed in MSCs isolated from other sources, e.g. bone marrow.

Based on the molecular biology, biophysics and pharmacology analysis above we propose that hHv1 channels are expressed in cMSCs isolated from human placenta. In order to demonstrate the physiological role of hHv1 in cMSC we applied the pharmacological inhibitor of the current, ClGBI. The interpretation of the physiological assays, such as mineralization, wound healing and migration requires the knowledge of the cytotoxic effects of these compounds. Based on the MTT assay we report here reduced viability of cMSCs in the presence 100 μM ClGBI. On the contrary, the results of the MTT assay were oblivious to the presence of 100 μM ClGBI in COS-7, a cell type known to lack hHv1 expression^46^. Thus, the effect of ClGBI on cell viability seems to be restricted to cells expressing Hv1. Inhibition of hHv1 may lead to increased cytotoxicity through a variety of mechanisms that include primarily the regulation of pH_i_ and consequently, that of pH_i_-dependent cytosolic processes, enzymes, and mitochondrial oxidative metabolism^58,59^. The exact molecular mechanisms of these effects of ClGBI are unclear, but may involve cell death as well, and this can be used to target cancer cells abundantly expressing hHv1^18^.

MSCs can differentiate into osteoblast^9,60^, chondroblast^9,60^, cardiomyocytes^2,60,61^, smooth muscle cells^62,63^, endothelial cells^64^ among the others^9,13,60,61^. Differentiation of MSC strongly depend on the tissue microenvironment^60,65,66^. Blair *at al*. developed a mesenchymal stem cell preparation using various matrices in bioreactors, and their results suggest that the secretion of matrix strongly depends on the active and passive transport of protons and the control pH_i_ and pH_o_^67^. In addition, there is evidence about the role of MSCs in pathological mineralization^68^. This motivated us to study the effect of pharmacological inhibition of hHv1 on mineralization and osteogenic differentiation. We found that mineral matrix production was significantly inhibited by the hHv1 inhibitor ClGBI in the concentration range of 60–100 μM. This latter concentration of ClGBI is ~5x the K_d_ for the inhibition of the hHv1 current which blocks ~85% of the current^39^. As 100 μM ClGBI also induces a significant reduction on the MTT production (Fig. 4), the interpretation of the results more complex. This must include the inhibition of the proton current, which may interfere with the optimal pH_i_ for various cytosolic processes including mRNA expression and enzyme activity of alkaline phosphatase and/or the regulation of osterix transcription^58,69^. Other issues to consider are the reduction of the cell number which may limit matrix formation. However, dying cells serve as the nucleus of bone formation^70^, and thus promote mineral matrix formation. The combination of these opposing effects, in our interpretation, may result in the very shallow dose-response of ClGBI on mineral matrix formation reported in this paper. The importance of these counterbalancing effects is also manifested in the reduced sensitivity of mineral matrix formation to the inhibition of hHv1 by ClGBI in the pathological mineralization model. Pathological mineralization was induced by P_i_, which is known to activate voltage-gated proton channels^29^. The role of hHv1 in pathological mineralization is consistent with the presence of hHv1 transcripts in vascular smooth muscle cells (data not shown, Varga and Meszaros, manuscript in preparation).

Inhibition of hHv1 may also influence mineral matrix production by regulating pH_o_ in the transcellular H^+^ transport model of osteogenesis^67^. According to that model transporters in the osteoblasts mediate acid uptake from bone matrix at the osteoblast “apical” membranes and acid extrusion at the “basolateral” membranes. hHv1 may also participate in the proton extrusion among other transporters (Na^+^/H^+^ exchanger, Cl^−^/H^+^ exchanger etc^71–73^.). When hHv1 is inhibited cells may not secrete sufficient H^+^ on the “basolateral” side and thus resorb H^+^ on the “apical” side thereby leading to reduced matrix formation and eventually cell death due to H^+^ accumulation in the cytosol. This hypothesis implies a basolateral localization of Hv1 in these cells, which is different from the proposed apical localization in other polarized cells (e.g., airways epithelial cells^74,75^). Thus, hHv1-dependent intra- and extracellular pH regulation may have important effects on bone metabolism and these data can be used to develop new therapeutic strategies to prevent pathological or ectopic mineralization via the modulation of hHv1.

Migration capacity MSCs is crucial for the execution of their biological functions. For example, MSCs must be recruited to the site of tissue damage, a key factor of their application in regenerative medicine^13,14^. On the other hand, migration of MSCs into tumors promotes tumor growth and metastasis formation by the secretion of cytokines, growth factors, chemokines and many other soluble molecules^76–86^. Moreover, MSCs migrated into the tumor negatively modulate anti-cancer immunity via their interaction with immune cells^87–89^. Ion transporters and channels in the plasma membrane participate in the regulation of cell motility^90–94^. There is also evidence for the contribution of hHv1 to cell migration, e.g. in immune cells^20,24^ and in glioblastoma^21^, breast cancer^33^, colorectal cancer^32^. Our results show that wound healing is reduced when hHv1 is inhibited by ClGBI. As the result of the wound healing assay is also sensitive to cell number and density, we have extended the data analysis to individual cell trajectories. The rose plots on Fig. 8C,D clearly show that ClGBI treatment reduced the displacement of cMSCs from origin, which was associated with a reduction of the average speed of the migration. The combination of these may, at least partially, explain the reduced wound-healing observed upon ClGBI treatment. Similar to our findings Ribeiro-Silva L. *et al*. has shown that migration of glioblastoma is inhibited by the hHv1 blocker ZnCl_2_^21^. The cellular mechanism for the reduced migration upon hHv1 block is unclear at this moment, but it may be secondary to the acidification of the cytosol^21,32,33^. This latter idea is consistent with our data: the effect of ClGBI on cell migration was not immediate, an ~8 h incubation was required for the manifestation of the reduced wound healing.

In summary, we have described that the hHv1 proton channel is functionally expressed chorion derived mesenchymal stem cells. hHv1 inhibition resulted in reduced cMSC migration capacity, which in turn, might be a useful tool to influence therapeutic use of cMSCs by restricting their mobility. Whether hHv1 block inhibits the “beneficial” cellular responses of cMSCs, e.g. differentiation into myocytes, or immunomodulation in autoimmune diseases, should be further investigated.

## Methods

### Ethical approval

The Regional and Institutional Ethics Committee of University of Debrecen, Debrecen, Hungary approved the study (license number DEOEC-RKEB-2946-2009). All experimental techniques were performed in accordance with the relevant guidelines and regulations listed in the ethical approval. Informed consent of the participants has been obtained according to the ethical approval.

### Isolation of chorion-derived mesenchymal stem cells (cMSCs),cell culture condition and induction of the mineralization

Isolation of the mesenchymal stem cells from the chorionic plate of placenta (cMSC) was carried out as previously described^47^. Conventional surface markers (CD73, CD90 positive and CD34, CD45 negative) were used to identify cMSCs. Cells were cultured in DMEM containing standard supplements (10% FBS, 50 U/mL penicillin, 50 µg/mL streptomycin, and 1% L-glutamine) at 37 °C in humidified atmosphere of 5% CO_2_.

For the induction of mineralization we used two different methods: the “classic inducing medium” was supplemented with dexamethasone (0.1 µM), ascorbic acid 2-phosphate (50 µg/mL), β-glycerophosphate (10 mM), and vitamin D_3_ (50 nM)^47^, the pathological mineralization was induced by inorganic phosphate (3 mmol/L; pathological inducing medium)^95^. Cells density (580 cells/mm^2^) was the same in all experiments. Cells were seeded in growth medium and allowed to attach/settle for 24 hours, then differentiation was initiated by adding osteogenic medium (Day 0).

### hHv1 transfection

HEK-293 cells were used to express hHv1 (a kind gift from Kenton Swartz, NIH, Bethesda, MD, USA). HEK-293 cells were transiently co-transfected with a plasmid encoding hHv1 and another plasmid encoding green fluorescent protein (GFP) at molar ratios of 1:5, by using Lipofectamine 2000 (Invitrogen) according to the manufacturer’s protocol, then they were cultured under standard conditions. Transfected cells were replated onto 35-mm polystyrene cell culture dishes (Cellstar, Greiner Bio-One Hungary Kft, Mosonmagyaróvár, Hungary). Channels were transiently expressed in HEK-293 cells 12–48 h after transfection. GFP-positive transfectants were identified by a Nikon TS-100 fluorescence microscope (AuroscienceKft, Budapest, Hungary) using bandpass filters of 455–495 nm and 515–555 nm for excitation and emission, respectively. More than 60% of the GFP-positive cells expressed the co-transfected ion channels. Currents were recorded 24-48 h after transfection.

### Total RNA extraction, reverse transcription, and reverse transcriptase PCR (RT-PCR) analysis

The total RNA was extracted from cMSCs using TRIzolate Reagent (UD-GenoMedKft., Debrecen, Hungary; URN0101) according to the manufacturer’s instruction. RevertAid H Minus First Strand cDNA Synthesis Kit (ThermoFisher Scientific, Waltham, MA, USA)) were used to synthesis cDNA. NAC (no amplification control) was produced without reverse transcriptase using all RNA sample by setting up the RT reaction as usual. PCR reaction contained the following reagents: 5 µL cDNA, 0.3 µL DreamTaq DNA Polymerase (Thermo Fisher Scientific, EP0703), 3 µL 10X DreamTaq Buffer (Thermo Fisher Scientific), 0.5 µL (10 mM) dNTP Mix, 10–10 pM forward (FW) and reverse (RV) primer, 10.2 µL Nuclease-free water. To control the effectiveness of RNA isolation, GAPDH RT-PCR was carried out using primers from cDNA Synthesis Kit. NTC (no template control) was generated using water as template. The primers for detection of hHv1 are collected into the Supplementary Table ST1.

## Electrophysiology and pharmacology

Electrophysiology measurements were carried out using patch-clamp technique in voltage-clamp mode. Whole-cell currents were recorded form cMSCs using an Axopatch 200B amplifier connected to a DigiData 1440 digitizer (Molecular Devices, Sunnyvale, CA, USA). Micropipettes were pulled from GC 150 F-15 borosilicatecapillaries (Harvard Apparatus Kent, UK) resulting in 3- to 5-MΩ resistance in the bath solution. The standard extracellular solution at pH_o_ = 7.4 contained 135 mM L-aspartic acid (Na^+^ salt), 6 mM MgCl_2_, 5.5 mM glucose, 20 mM HEPES. The extracellular solution at pH_o_ = 6.4 contained 60 mM L-aspartic acid (Na^+^ salt), 6 mM MgCl_2_and 80 mM MES (titrated with NaOH). The measured osmolality of the extracellular solutions was about 300 mOsm/L. The pipette solution contained 90 mM L-aspartic acid (Na^+^ salt), 80 mM MES, 6 mM MgCl_2_, 3.3 mM glucose at pH = 6.18 (titrated with NaOH). The solutions did not contain potassium to exclude the contamination of the records by potassium currents. Zn^2+^ was also used in NMDG (N-Methyl-D- Glucamine)-based extracellular solution at pH_o_ = 7.4. It contained 75 mM NMDG (N-Methyl-D- Glucamine), 3 mM MgCl_2_, 15 mM glucose, 180 mM HEPES (titrated with HCl).

Guanidine derivate 5-chloro-2-guanidinobenzimidazole (CIGBI; Sigma-Aldrich Kft. Budapest, Hungary, S517038) and Arachidonic acid (AA; Sigma-Aldrich, A3611-100MG) stock solutions (concentration of the stocks was 30 mM) were prepared in DMSO.

## Examination of the mineralization

Calcium deposition was assayed with Alizarin Red S staining. The staining was carried out as previously described^47^. After staining cell monolayers were photographed, and for the quantification Alizarin Red S-calcium complexes were extracted from the stained cultures with 10% cetylpyridinium chloride (CPC) in 10 mM sodium phosphate buffer (pH 7.7). The optical density of the color was determined at 540 nm using spectrophotometer in each well. DMSO treatment was used as control. Data obtained with DMSO control was statistically identical. Normalized mineralization was calculated as A/A_DMSO_ where A and A_DMSO_ are the absorbance’s a given sample and for differentiated cells in the presence of DMSO control, respectively.

## MTT Assay

The most common method for the determination of cell viability is the MTT reduction assay. The measurement based on the conversion of MTT (Thiazoly Blue Tetrazolium Bromide, Sigma-Aldrich, M2128-1G) to formazan in the cell culture, which was carried out according to the manufacturer’s instructions. Cell viability was presented as a percentage of control (Normalized OD). DMSO treatment were used as vehicle controls, results were normalized to non-differentiated cells in the presence of DMSO.

### Monitoring of cell migration using fluorescence labeling and wound healing assay

cMSC cells were cultured in the presence or absence of CIGBI for 41.3 hours in 96-well Oris Pro Cell Migration Assay plate (Platypus Technologies LLC, Madison, WI, USA). Each well in the assay plate contained dissolving bio-compatible gel droplet which formed cell free zone in the center of the wells. Immunofluorescence staining of MSCs was made using Cell Tracker CM-Dil (vital membrane dye C7000, Invitrogen, Carlsbad CA, USA). Images acquired by Opera Phenix High Content Confocal System (PerkinElmer, Waltham, MA, USA). Images of the Alexa-561 channel was collected at 8 μm of Z image plane using a 5× air objective (NA: 0.16) to monitor multigenerational tracking of cellular movements. 9 fields were acquired with 5% overlap that were covered the entire well and laser-based autofocus was performed at each imaging position. Time series measurements were created for 41.3 hours using 20 minutes interval between each individual measurement. The primary data were analyzed by Harmony 4.8 software (PerkinElmer) according to the analysis workflow of Migration - Confluency and Cell Tracking Ready to Made Solutions (http://www.perkinelmer.com/product/harmony-4-2-office-hh17000001). Open areas were determined and separated from cell layer by machine learning user defined training methods on the basis of Alexa-561 staining intensity.

The raw tracking data of cells were set up into a database, included induvial cell identifiers, coordinates and time points in all wells and frames of all cells, the database was exported to csv text file. The analyses of exported data were implemented in custom-written MATLAB scripts (The MathWorks, Natick, MA). The initial cell free circular region of the center of the wells was used as a spatial filter for constructing the cell trajectories. All cell trajectories within the initial cell free region longer than 10 steps were collected and analyzed for calculating the step sizes and visualizing the cellular migration. Step size data were presented as probability density histograms, for statistical comparisons, the Kolmogorov-Smirnov test was used. For visualizing the effect of cell migration upon treatment, wind-rose plots depicting migratory tracks of ten randomly chosen individual migrating cells with step length of twenty were shown from control and treated samples.

## Data analysis

For multiple comparisons One Way Analysis of Variance on Rank test were used, the difference between samples collected on two different day were tested by t-test or Mann-Whitney Rank Sum Test. Statistical significance was concluded at *p < 0.05 (*indicates statistical difference). Determination of the threshold potential was the following: For the current recorded by ramp protocol, first off line leak correction was performed (i.e. a straight line was fitted to the points recorded in the first 200 ms of the 1 sec long ramp protocol (protocol ran from −60mV up to +150 mV). The parameters of this line were used to calculate the leak.). Then the mean and the SD values were determined in the first 100 ms. When 20 consecutive data points were above 2xSD value than that time in millisecond was associated to the threshold value. Finally, this threshold time value was converted to the appropriate voltage, i.e. membrane potential which is the threshold potential itself.

For current recorded using the I-V protocol, (voltage ran from −80 mV up to +100 mV with 10 mV increments) the process was very similar. Namely, the mean and the SD values were determined using the first 6 data points (i.e. between −80 mV and −30 mV). The threshold potential was associated to the current value, which was above 5xSD.

## Supplementary information

Supplementary information.
