# Supplementary material for: The voltage-gated proton channel hHv1 is functionally expressed in human chorion-derived mesenchymal stem cells
Source: Sci Rep. 2020 Apr 28;10:7100. doi: 10.1038/s41598-020-63517-3 (PMC7188850; doi:10.1038/s41598-020-63517-3)
Supplement: Supplementary file 1 — Supplementary information. [file 41598_2020_63517_MOESM1_ESM.pdf]

SUPPLEMENTARY MATERIAL

The voltage-gated proton channel hHv1 is functionally expressed in human chorion-derived mesenchymal stem cells

Beáta Mészáros[a,c], Ferenc Papp[a], Gábor Mocsár[a], Endre Kókai[b], Katalin Kovács[b,c], Gábor Tajti[a] and Gyorgy Panyi[a,c,*]

[a] Department of Biophysics and Cell Biology, Faculty of Medicine, University of Debrecen, Life Science Building, Debrecen, Egyetem ter 1. Hungary, H-4032

[b] Department of Medical Chemistry, Faculty of Medicine, University of Debrecen, Life Science Building, Debrecen, Egyetem ter 1. Hungary, H-4032

[c]MTA-DE Cell Biology and Signaling Research Group, University of Debrecen, Life Science Building, Debrecen, Egyetem ter 1. Hungary, H-4032

*Corresponding author:

Gyorgy Panyi
Department of Biophysics and Cell biology
University of Debrecen

1 Egyetem ter
Life Science Building, Room 2.305
4032, Debrecen,
Hungary

phone: (+36)(52) 258-603 (Dept. secretary)
email: panyi@med.unideb.hu

Supplementary figures

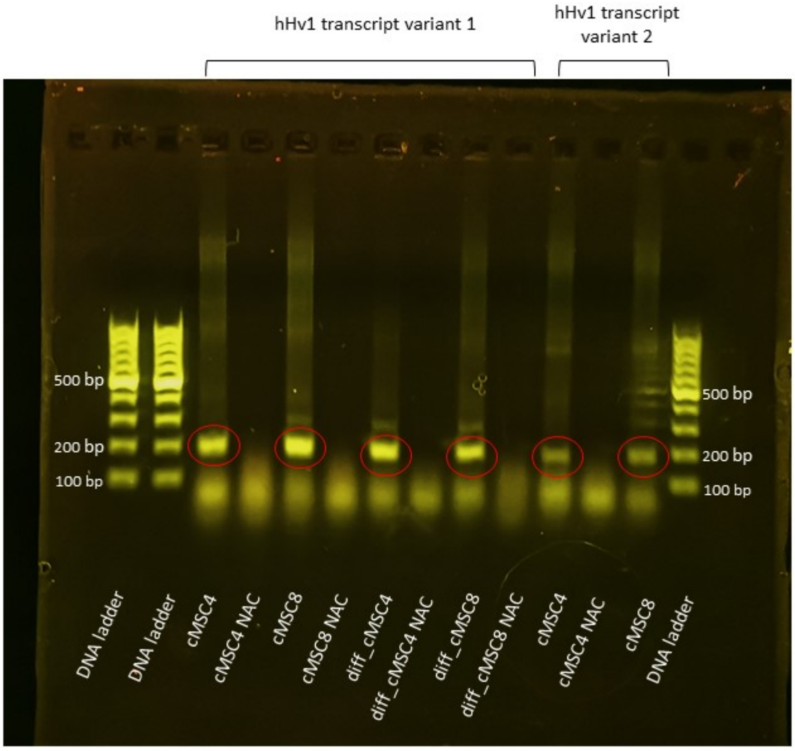

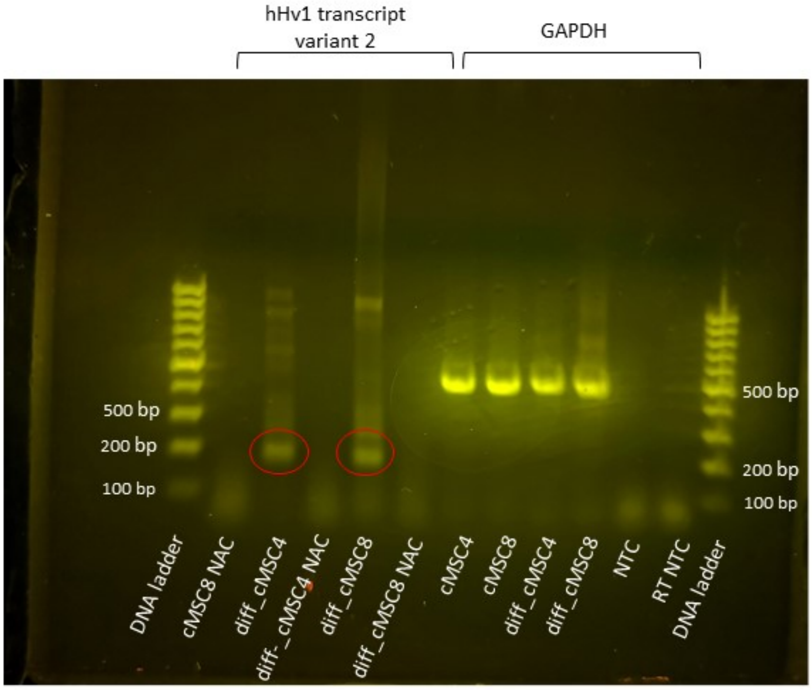

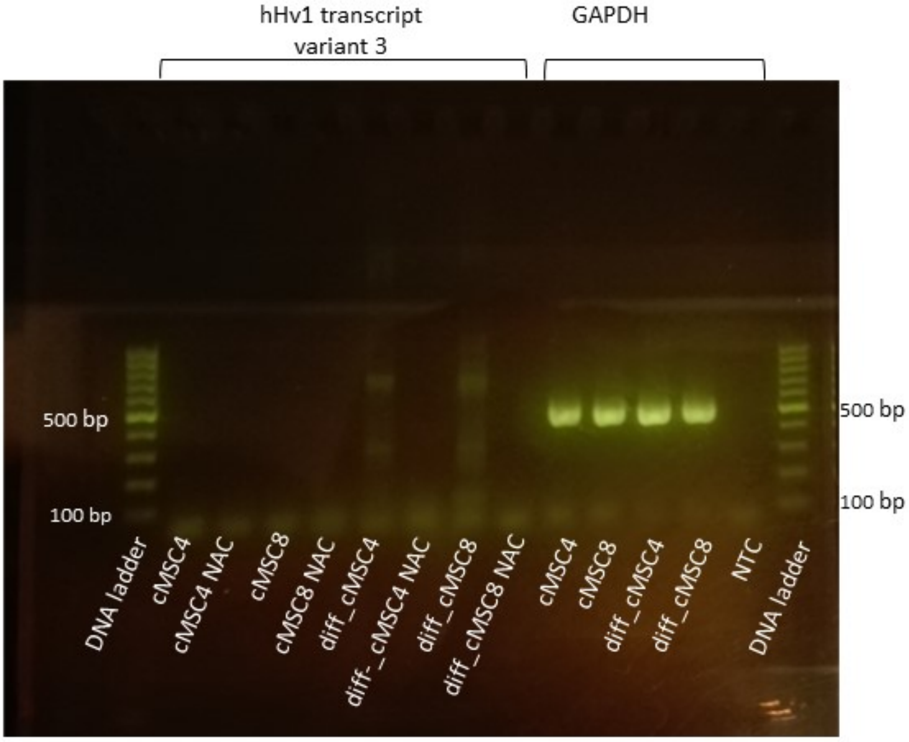

Supplementary figure S1. hHv1 transcript variants 1 and 2 are detected in differentiated and non-differentiated cMSCs from placenta donor 4 and 8

Identification of hHv1 mRNA was carried out by means of RT-PCR, using intron spanning primers to confine the distinct transcript variants. Red circles on the gel electrophoresis photos highlight the presence of the transcripts of hHv1 variant 1 (PCR product is 190 bp) and hHv1 variant 2 (PCR product is 183 bp) in cMSCs isolated from placenta donors 4 (shown as cMSC4 in the figure) and 8 (shown as cMSC8 in the figure), as indicated. We did not detect the transcript variant 3 (missing 568 bp long PCR product). The osteogenic differentiated cells from the same donors were labelled as diff_cMSC4 and diff_cMSC8. The PCR was validated using GAPDH and NTC (non-template control) controls (right set). The NAC bands (no amplification control) were obtained in the absence of the reverse transcriptase enzyme. NAC controls showed that genomic hHv1 DNA was not amplified in the positive samples.

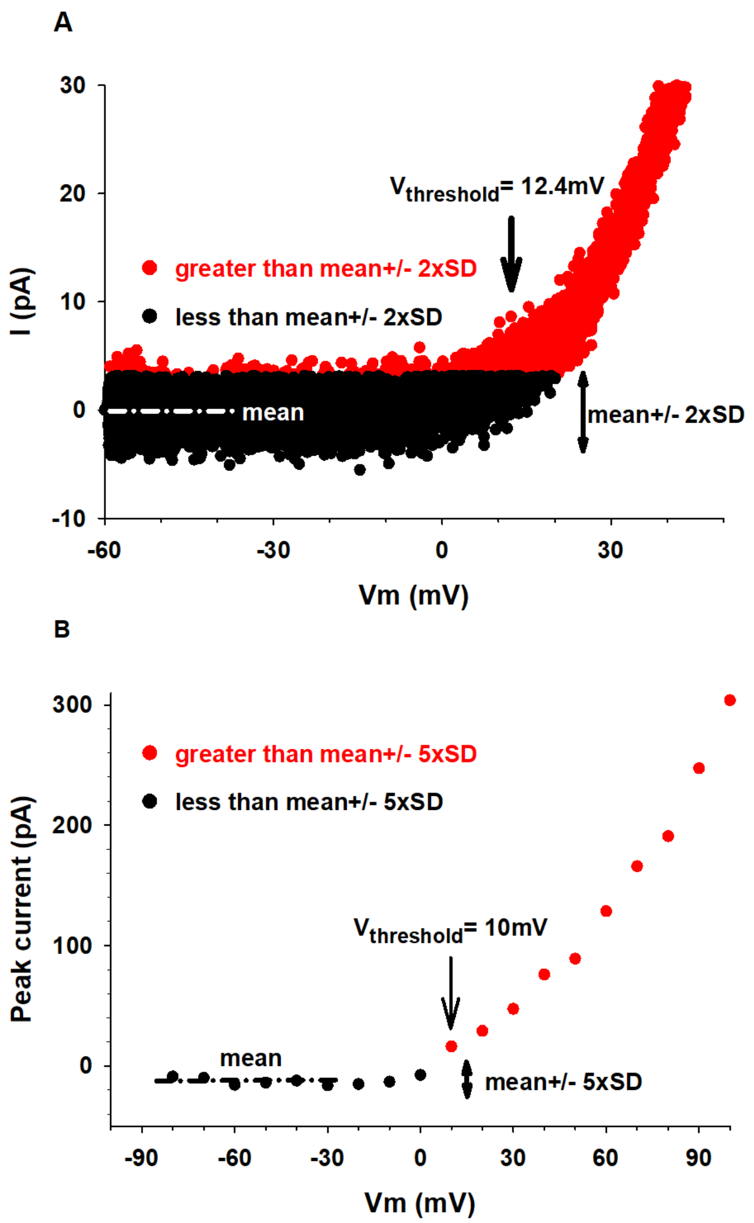

Supplementary figure S2. Determination of the threshold potential (V_{thr}) in cMSCs

A, Determination of the V_{thr} using ramp protocol in cMSC10. The cell was held at –60 mV holding potential, the membrane potential was changed at a constant rate from –60 mV to +150 mV in 1000 ms, and the whole-cell currents were recorded using pH_o=7.4 bath and pH_i=6.18 pipette filling solutions. First off line leak correction was performed. Then the mean current (white dot-dash line) and the SD values (vertical solid black arrow) were determined in the first 100ms of the ramp corresponding to the range of [–60 mV and –39 mV] membrane potentials, where the current is not activated. When 20 consecutive data points were above mean+2xSD value than that time in millisecond was associated to the threshold value. Finally, this threshold time value was converted to the appropriate voltage and displayed on the abscissa. B, Determination of the V_{thr} using I-V protocol. Whole-cell patch-clamped cMSC cell was held at –80 mV, test potentials were delivered from –80mV up to +100mV with 10mV increments every 20s. The peak currents were determined at the end of the depolarizing pulses and plotted as a function of the test potential. The mean current (black dotted and dashed line) and the SD values (vertical solid black arrow) were determined using the first 6 data points (i.e. between –80mV and –30mV), where the current is not activated. The threshold potential was associated to the test potential where the current was above mean+5xSD.

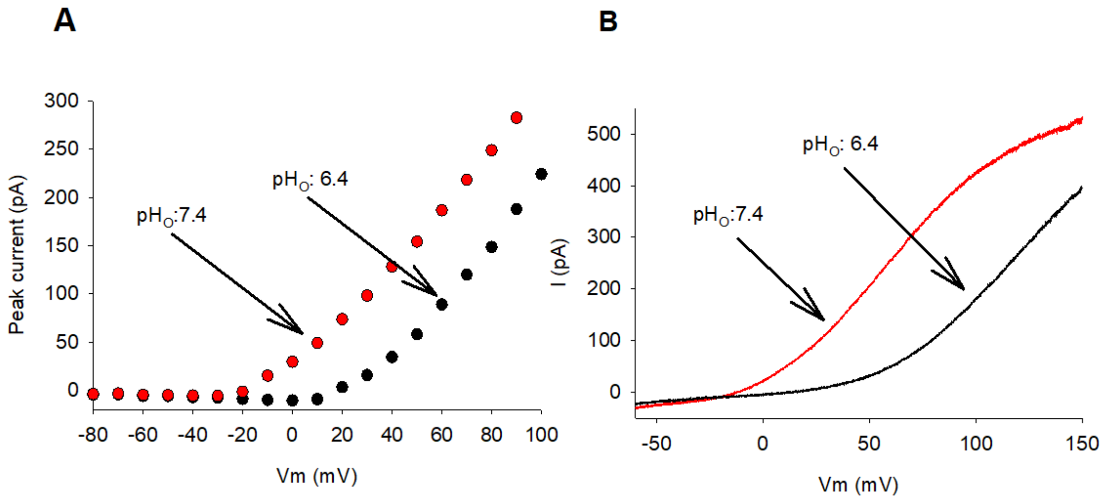

Supplementary figure S3. Voltage-dependent gating of hHv1 modulated by pH in transfected HEK 293.

Changing pH_o by one unit shifts the voltage-dependence of Hv1 channels by about 40 mV toward more negative voltages. A, Current- voltage relationships were constructed from outside-out patch records. The patch was excised from a HEK-293 cell expressing hHv1. The patch was held at −80 mV, test pulses were delivered ranging from −80 mV to +100 mV with 10 mV increments every 20 s. Peak currents were determined at the end of the depolarizing pulses at the indicated test potentials. Red and black circles indicate the magnitude of the currents at extracellular pH_o=7.4 and pH_o=6.4, respectively. The pipette was filled with pH_i=6.18 solution. The voltage shift in the threshold potential caused by changing the pH_o by 1 unit was ~40 mV. B, Currents evoked by voltage ramps at different pH_o. The red and black traces correspond to records at pH_o=7.4 and pH_o=6.4, respectively, as indicated also by the arrows. The holding potential was −60 mV, the membrane potential was changed at a constant rate from −60 mV to +150 mV in 1000 ms, every 20 s. The difference between the threshold potential at pH_o=7.4 at pH_o=6.4 was 48.7 mV in this record (pH_i=6.18 for the pipette filling solution).

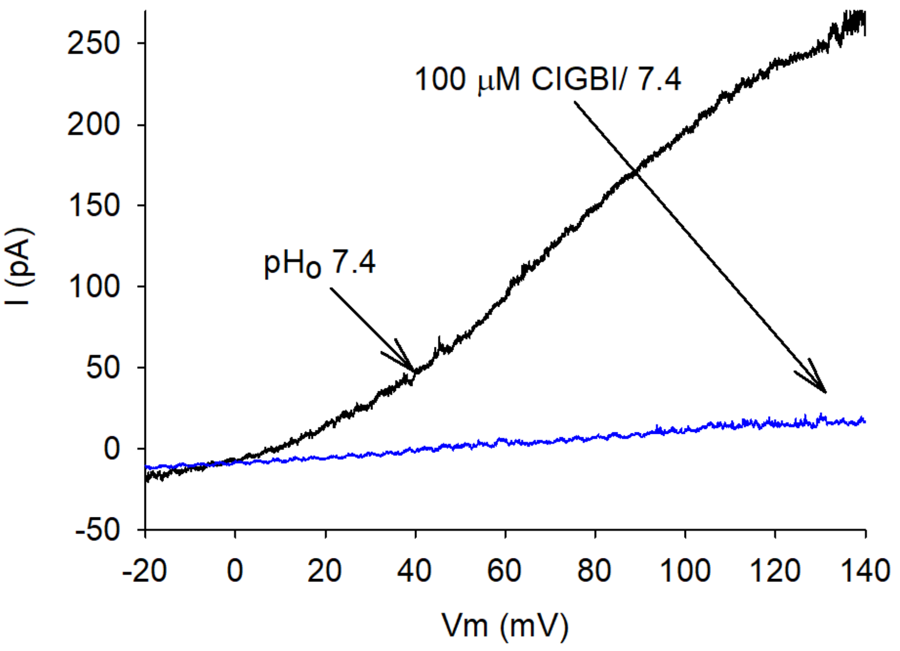

Supplementary figure S4. Block of the current by ClGBI in HEK-293 expressing hHv1

Currents were recorded in outside-out patch configuration. The patch was excised from a HEK-293 cell expressing hHv1. The holding potential was –60 mV, the membrane potential was changed at a constant rate from –60 mV to +150 mV in 1000 ms, every 20 s (the figure shows the currents from –20 to +140 mV to improve clarity). The control trace (black) was recorded using pH_o=7.4 extracellular solution and pH_i=6.18 pipette filling solution. The blue trace (arrow) indicates the record in the presence of 100 μM ClGBI in the extracellular solution at pH_o=7.4. 100 μM ClGBI inhibited 93.7% of the current at +150 mV at pH_o=7.4.

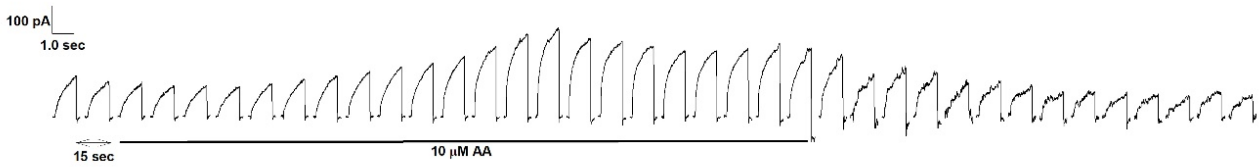

Supplementary figure S5. Reversible enhancement of the hHv1 current by AA.

Currents were measured in the whole-cell configuration of patch-clamp in a cMSC. Depolarizing test pulses (+100 mV, 1 s in duration) were applied every 30 s from holding potential –80 mV. The black bar indicates the period of the application of 10 μM AA using a perfusion system.

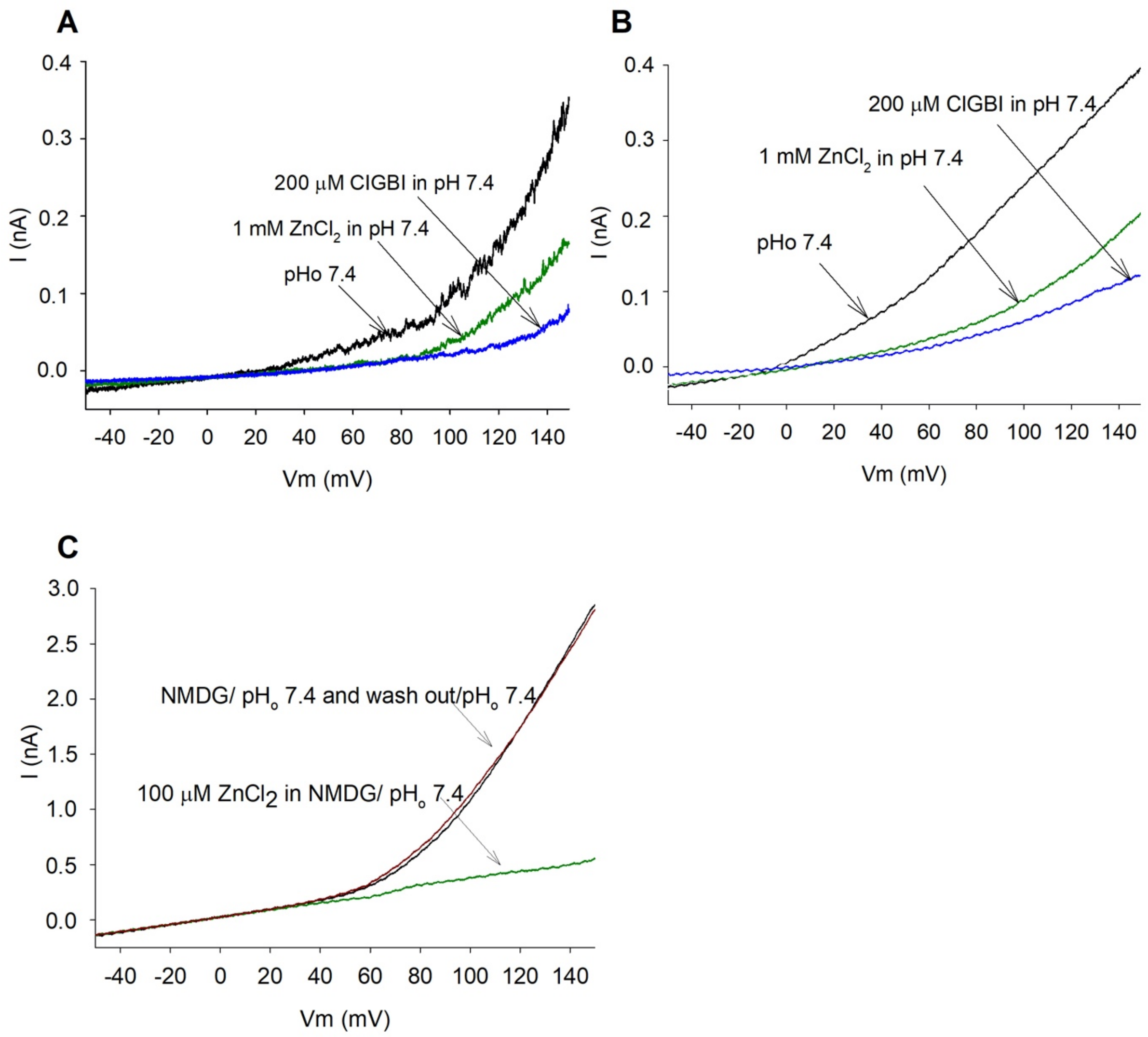

Supplementary figure S6. The effect of Zn^{2+} in aspartate-based and NMDG-based extracellular solution in a cMSC and transfected HEK 293 cells

A, Inhibition of the hHv1 current by $ZnCl_2$ in cMSC4 in the aspartate-based extracellular solution. Whole-cell currents were evoked by voltage-ramps at pH_o=7.4. The holding potential was –60 mV, the membrane potential was changed at a constant rate from –60 mV to +150 mV in 2000 ms, every 20 s. The control record was obtained at pH_o=7.4 (black). 81 % and 50% of the current at +150 mV was blocked using 200 µM ClGBI (blue) and 1mM $ZnCl_2$ (green). Both inhibitors were dissolved at pH_o=7.4. B, Inhibition of the current in hHv1 transfected HEK cell by ClGBI and Zn^{2+} in the aspartate-based extracellular solution. 200 µM ClGBI (blue) and 1mM $ZnCl_2$ (green) was applied using the same protocol as in A except in outside-out configuration of patch-clamp.. Approximately 95 % and 50% of the current at +150 mV was blocked using 200 µM ClGBI and 1mM $ZnCl_2$, respectively. C, Inhibition of the current in hHv1 transfected HEK cell by ClGBI and Zn^{2+} in the NMDG-based extracellular solution. Control records were obtained at pH_o=7.4 (black) and in the presence of 100 µM Zn^{2+} pH_o=7.4 (green) in whole cell configuration using the same protocol as in A. 90% of the current measured at +150 mV was blocked by 100 µM Zn^{2+} at pH_o=7.4. Wash-out of the effect of Zn^{2+} was complete at pH_o=7.4 (dark red). NMDG supplemented with 100 µM Zn2+ inhibited 92 ± 5% of the current (n=5), which agrees well with the literature (estimated K_d = 1.9 µM). The composition of the NMDG-based extracellular solution is in the Materials and Methods. Zn^{2+} at 10x higher concentration (1 mM) blocked only partially the hHv1 current either in cMSCs(A) or in transfected HEK (B) when the aspartate-based extracellular recording solution was used rather than the NMDG-based one.

```
9     ---------------------------------------TATGCTGCCATGGTATTCCAC  29
                                             |||||||||||||||||||||
361   ATCCTGGACCTGAAGATCATCCAGCCCGACAAGAATAACTATGCTGCCATGGTATTCCAC  420

30    TACATGAGCATCACCATCTTGGTCTTTTTTATGATGGAGATCATCTTTAAATTATTTGTC  89
      ||||||||||||||||||||||||||||||||||||||||||||||||||||||||||||
421   TACATGAGCATCACCATCTTGGTCTTTTTTATGATGGAGATCATCTTTAAATTATTTGTC  480

90    TTCCGCCTGGAGTTCTTTCACCACAAGTTTGAGATCCTGGATGCCGTCGTGGTGGTGGTC  149
      ||||||||||||||||||||||||||||||||||||||||||||||||||||||||||||
481   TTCCGCCTGGAGTTCTTTCACCACAAGTTTGAGATCCTGGATGCCGTCGTGGTGGTGGTC  540

150   TCATTCATCCTCGACATTGTCCTCCTGTTCCAGGAGCACCAGTTTGAGGCTCTGGGCCTG  209
      ||||||||||||||||||||||||||||||||||||||||||||||||||||||||||||
541   TCATTCATCCTCGACATTGTCCTCCTGTTCCAGGAGCACCAGTTTGAGGCTCTGGGCCTG  600

210   CTGATTCTGCTCCGGCTGTGGCGGGTGGCCCGGATCATCAATGGGAA               256
      |||||||||||||||||||||||||||||||||||||||||||||||
601   CTGATTCTGCTCCGGCTGTGGCGGGTGGCCCGGATCATCAATGGGATTATCATCTCAGTT  660
```

```
YAAMVFHYMSITILVFFMMEIIFKLFVFRLEFFHHKFEILDAVVVVVSFILDIVLLFQEHQFEALGLLILLRLWRVARIING
YAAMVFHYMSITILVFFMMEIIFKLFVFRLEFFHHKFEILDAVVVVVSFILDIVLLFQEHQFEALGLLILLRLWRVARIING
```

Supplementary figure S7. Determination of His140 and His193 coding mRNA sequences in cMSCs using RT-PCR and DNA sequencing

Two His residues in hHv1 are responsible for high-affinity Zn^{2+} binding, His140 and His193. We designed primers for a short, 281 bp long sequence of HVCN1 transcript variants, which contained the two His coding codons and we amplified this 281 bp long DNA segment and determined the sequence of the PCR product by sequencing. The align of the original (gene bank, three variants aligned) and amplified DNA sequences are shown on the top panel whereas the coded amino acid sequences are compared on the bottom panel. The His residues are in red color, and the homology was 100%.

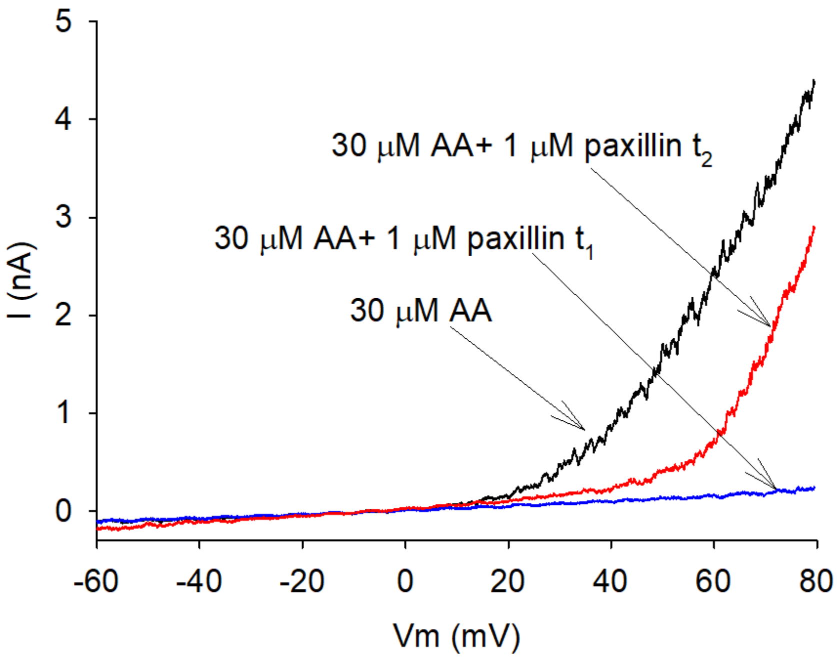

Supplementary figure S8. KCa1.1 and hHv1 currents are simultaneously present in cMSCs.

Whole-cell currents were evoked in a cMSC by voltage-ramps. The holding potential was −100 mV, the membrane potential was changed at a constant rate from −100 mV to +80 mV in 500 ms, every 20 s (the figure shows the currents from −60 to +80 mV to improve clarity). The currents were recorded using the standard extracellular solution at pH_o=7.4 and a potassium-containing pipette filling solutions at pH_i=7.4. The composition of the intracellular solution was (in mM) potassium aspartate 134, $MgCl_2$ 9.5, $CaCl_2$ 1, EGTA 10, HEPES 10 (pH 7.4 adjusted with KOH). Records were obtained in the presence of 30 μM AA (black). This current was inhibited almost fully (96.5% of the current at +80 mV) by 1 μM paxillin (blue, t_1 time point). In the continuous presence of 1 μM paxillin and 30 μM AA a whole cell current with different activation threshold reappeared at t_2 time point (red). t_1= 0 min., upon application of paxillin,, t_2= 5 min. We propose that AA-induced current appearing when KCa1.1 was fully inhibited represents the AA-activated hHv1 current. The triple combination of 30 μM AA+ 1 μM paxillin + ClGBI was not possible, the recording configuration become unstable after prolonged AA exposure.

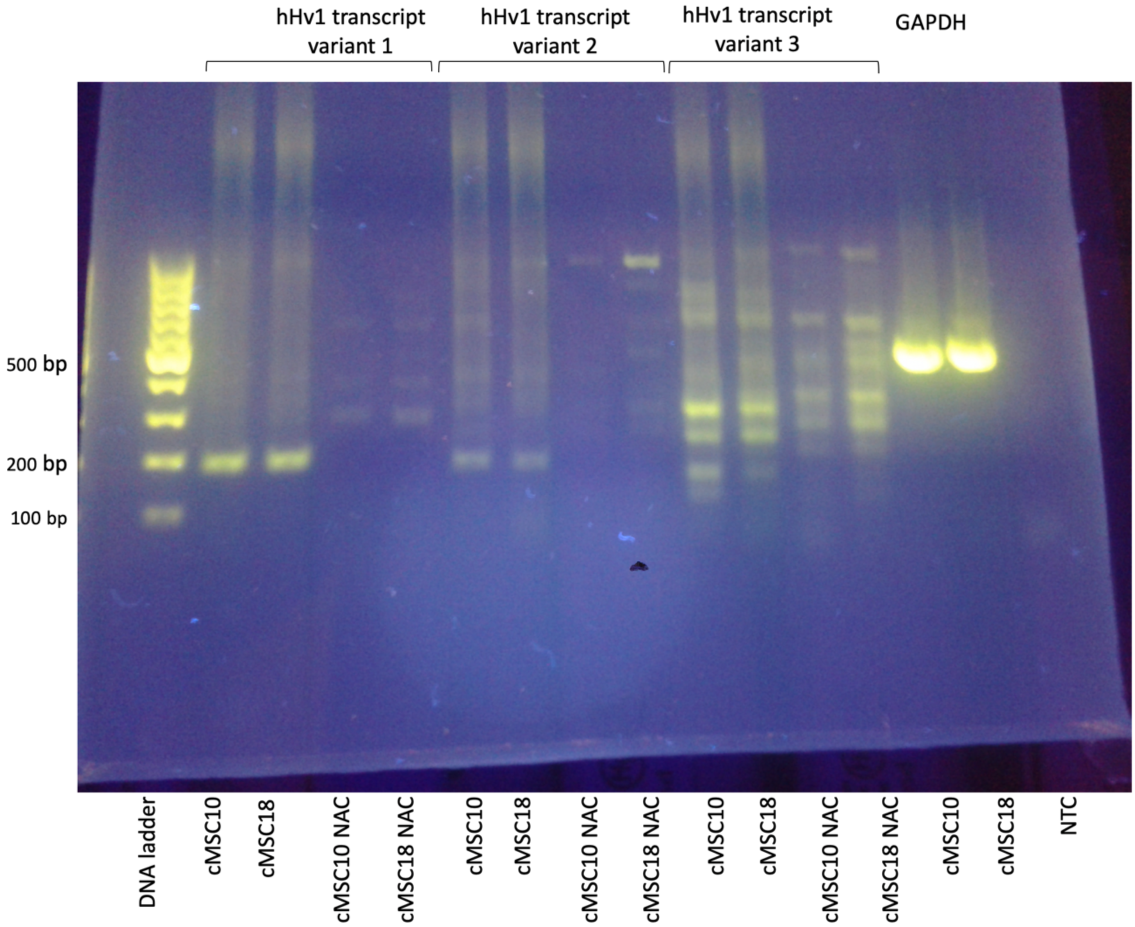

Supplementary figure S9. Full length gel photo corresponding to Fig. 1.

Please see details there. The PCR was validated using GAPDH and NTC (non-template control) controls (right set). The NAC bands (no amplification control) were obtained in the absence of the reverse transcriptase enzyme. cMSC10 and cMSC18 correspond to different donors.

Target	Primer	Primer sequences 5'- 3'	Size of PCR product (bp)	PCR profile
NM_001040107.1 HVCN transcript variant 1	hHv1a_FW	CGTACGAGTTGGCCCGGAG	**190**	95 °C 1 m<br>95 °C 30 s }<br>62 °C 30 s } 35X<br>72 °C 1 m }<br>72 °C 5 m
	hHv1a_RV	TGCGGGTGACTGCCTTTTC		
NM_032369.3 HVCN1 transcript variant 2	hHv1b_FW	CTGCTCTGAGGCTCCCAGTC	**183**	
	hHv1b_RV	TGACTGCCTTTTCGTCCCAG		
NM_001256413.1 HVCN1 transcript variant 3	hHv1c_FW	GACTTCAGAGCTGGGCCTG	**568**	
	hHv1c_RV	AGTGGAATACCATGGCAGCA		
HVCN1 homologue sequence of the 3 different transcript variant	hHv1_FW	GGACCTGAAGATCATCCAGCCCGACA	**281**	
	hHv1_RV	TCCCATTGATGATCCGGGCCACCC		

Supplementary table ST1. Sequences of the primers and the PCR condition

The first column contains the target of the primers, the second contain the name of primers, the 3rd column describe the primer sequences from 5' to 3', the next column shows the size of PCR products, and the last column contain PCR profile or condition.
